# A Review on Terahertz Technologies Accelerated by Silicon Photonics

**DOI:** 10.3390/nano11071646

**Published:** 2021-06-23

**Authors:** Jingya Xie, Wangcheng Ye, Linjie Zhou, Xuguang Guo, Xiaofei Zang, Lin Chen, Yiming Zhu

**Affiliations:** 1 Terahertz Technology Innovation Research Institute, Terahertz Spectrum and Imaging Technology Cooperative Innovation Center, Shanghai Key Lab of Modern Optical System, University of shanghai for Science and Technology, Shanghai 200093, China; xiejy@usst.edu.cn (J.X.); 193720547@st.usst.edu.cn (W.Y.); xgguo@usst.edu.cn (X.G.); zangxf_sjtu@163.com (X.Z.); linchen@usst.edu.cn (L.C.); 2 State Key Laboratory of Advanced Optical Communication Systems and Networks, Department of Electronic Engineering, Shanghai Jiao Tong University, Shanghai 200240, China; ljzhou@sjtu.edu.cn; 3 SJTU-Pinghu Institute of Intelligent Optoelectronics, Pinghu 314200, China

**Keywords:** terahertz technologies, silicon photonics, hybrid silicon photonics

## Abstract

In the last couple of decades, terahertz (THz) technologies, which lie in the frequency gap between the infrared and microwaves, have been greatly enhanced and investigated due to possible opportunities in a plethora of THz applications, such as imaging, security, and wireless communications. Photonics has led the way to the generation, modulation, and detection of THz waves such as the photomixing technique. In tandem with these investigations, researchers have been exploring ways to use silicon photonics technologies for THz applications to leverage the cost-effective large-scale fabrication and integration opportunities that it would enable. Although silicon photonics has enabled the implementation of a large number of optical components for practical use, for THz integrated systems, we still face several challenges associated with high-quality hybrid silicon lasers, conversion efficiency, device integration, and fabrication. This paper provides an overview of recent progress in THz technologies based on silicon photonics or hybrid silicon photonics, including THz generation, detection, phase modulation, intensity modulation, and passive components. As silicon-based electronic and photonic circuits are further approaching THz frequencies, one single chip with electronics, photonics, and THz functions seems inevitable, resulting in the ultimate dream of a THz electronic–photonic integrated circuit.

## 1. Introduction

Terahertz (THz) radiation, which occupies the frequency gap between the infrared and microwaves, typically referred to as the frequencies between 0.1 THz and 10 THz, becomes increasingly important for many applications such as data communications, biology, medical sciences, sensing, and imaging [1,2,3]. THz radiation penetrates deep into nonpolar and nonmetallic materials such as paper, plastic, clothes, wood, and ceramics that are usually opaque at optical wavelengths. These are also common packaging materials. Hence, THz imaging devices have been used for nondestructive testing in hidden object detection which is harmless for humans in contrast to X-rays. Furthermore, the THz region is also crowded by innumerable spectral features associated with fundamental physical processes such as rotational transitions of molecules, large-amplitude vibrational motions of organic compounds, lattice vibrations in solids, intraband transitions in semiconductors, and energy gaps in superconductors. THz applications exploit these unique characteristics of material responses to effectively detect and identify weapons, explosives, and chemical and biological agents concealed underneath various covering materials. Although THz technologies have great potential in a variety of applications, the lack of efficient and compact sources and detectors led to the THz band being called the “THz gap”.

This technological gap has been rapidly diminishing during the last two decades. While microwave technologies are encroaching up from the low-frequency side, photonics techniques are particularly attractive for both THz generation and detection, especially when broadband tunability of the THz frequency is required [2,3,4,5]. However, early photonics-based THz research has largely relied on the use of bulky equipment which is costly and requires high driving powers for efficient operation. Assembling all these components into useful products further reduces the reliability and mass-manufacturability of the device. Hence, a key goal is to be able to generate, modulate, and detect THz waves at the chip level. In addition, since the acquisition and analysis of THz signal need electrical units, the ideal scheme would be to integrate all the optical and electrical units and leverage chip-scale integration experienced in semiconductor-based photonics and electronics.

Now, there are signs that this might not be just a dream. Silicon photonics is compatible with industrial CMOS technologies, which enables high-volume production for a low cost per device [6,7]. Furthermore, it provides high index contrast between the core and cladding of the waveguide, allowing large-scale and high-density integration. Hence, just as in the world of III–V microelectronics, there is a growing desire to use silicon photonics for integrated THz applications to leverage cost-effective and high-volume fabrication. To the best of our knowledge, there is no overview of the current state of the research and achievements in this field. We therefore believe it would be helpful to list and discuss the latest THz techniques based on silicon photonics.

The paper is organized as follows. In Section 2, we list the main methods that are used to enable photonics-based THz generation and explain how bulky free-space or fiber-based off-the-shelf components can provide this functionality. In Section 3, we explain the main photonics-based THz detection methods likewise. Since the current THz system can be divided into two categories: pulsed light and continuous light, the above sections are introduced respectively in terms of THz broadband pulses and continuous waves (CWs). We then provide an overview of the silicon photonics or hybrid silicon photonics that has already been used in the THz applications. In Section 4, we introduce the current applications of silicon photonics technology in THz generation, detection, phase modulation, and intensity modulation. In addition, we also provide a brief overview of THz silicon dielectric waveguide devices based on similar silicon photonics technology. Finally, in Section 5, we conclude the paper with an outlook for this field, exploring whether THz-wave applications could be the next key application of silicon photonics after datacenter interconnects and telecommunications.

## 2. Generating THz Waves through Photonic Approaches

THz is at the intersection of the traditional microwaves and the far-infrared region, which means that electronics, optics, or a combination of both can be used to generate THz waves [8,9]. Optical generation of THz radiation falls into two general categories. The first category of approaches involves generating an ultrafast photocurrent in a photoconductive switch or semiconductor [10,11]. In the second category, THz waves are generated by nonlinear optical effects such as optical rectification [12,13], difference-frequency generation [14], or optical parametric oscillation [15]. According to the type of lasers used to generate THz signals, these technologies can be divided into two types in another dimension: pulsed and continuous wave (CW) THz technologies. Pulsed THz radiation provides wide bandwidth and can achieve very fast measurement. However, its frequency resolution is relatively limited, usually in the order of several GHz. CW THz is generated by mixing two laser beams of different frequencies, which has the advantages of precise frequency tuning and high resolution and is more suitable for high-speed THz communication.

### 2.1. Generation of Broadband THz Pulses

Since the early 1990s, the introduction of mode-locked femtosecond lasers has significantly expanded the use of THz time-domain spectroscopy (THz-TDS) [16,17,18,19,20,21]. Sub-picosecond THz pulses can be generated from a biased photoconductive (PC) antenna or nonlinear media excited by femtosecond laser pulses. Low-temperature-grown of gallium arsenide (LT-GaAs) material is often used in a PC antenna [22]. Femtosecond lasers are an essential tool to study ultrafast phenomena on a sub-picosecond time scale. The temporal resolution of such studies is primarily determined by the optical pulse duration. The time representation of typical broadband THz radiation takes the shape of a single-cycle pulse. In addition to short pulse duration, another crucial property of ultrashort optical pulses is that the peak intensity can be extremely high because all of the optical energy is concentrated in such a short period. A PC antenna is an electrical switch exploiting the increased electrical conductivity of semiconductors when they are exposed to light. The photoconductivity results from an increase in the number of free carriers (electrons and holes) generated by photons. The switching action in the PC antenna with a bias voltage occurs in the subpicosecond time scale. The switch-on time is a function of the laser pulse duration, and the switch-off time is mainly determined by the photoexcited carrier lifetime in the semiconductor substrate of the antenna; therefore, in addition to a short laser pulse duration, a short carrier lifetime is a vital property for ultrafast photoconductive switching. Another approach is to use the media with a large second-order nonlinear coefficient to generate THz pulses by nonlinear optical effects, such as gallium arsenide (GaAs) [23], zinc telluride (ZnTe) [24], diethylaminosulphur trifluoride (DAST) [13], and lithium niobate (LiNbO_3_) [25,26]. Although the bandwidth and performance of the THz-TDS depend on the experimental conditions, an overview of several THz generation techniques has been reviewed by Jens Neu and Charles A. Schmuttenmaer to compare different THz pulsed emitters [27]. The key elements of the wide spectrum and high-efficiency THz pulse generation are mode-locked lasers, photoconductive antennas, or nonlinear media, which eventually need to be integrated to reduce the footprint and energy consumption, etc.

### 2.2. Generation of THz Continuous Waves

Unlike the generation of broadband THz pulses that mainly rely on ultrafast optical technology, the CW THz radiation has a long history, and many different kinds of technical schemes are available. In the submillimeter-wave range, electronic sources such as Gunn diodes [28], impact avalanche transit-time diodes [29], and resonant tunneling diodes [30] are possible candidates as generators. However, their performance degrades at higher frequencies [31]. A common method to overcome the frequency limitations is using frequency multipliers based on Schottky diodes [32,33]. Other common solutions for generating THz high output power are vacuum electronic devices, such as backward-wave oscillators (BWOs) which are based on the interaction of electron beam with a slow wave structure [34,35]. The operating frequency of the BWOs can be electronically tuned from sub-THz to 1.5 THz frequency range [36]. Free electron lasers produce coherent radiation from high-speed electrons moving freely through a periodic magnetic structure [37]. They are able to emit very high powers and are tunable in a wide frequency range, but they are very large and expensive. Gas lasers based on optically pumping polar molecules by infrared lasers are one of the most powerful CW THz sources [38]. They typically have output power up to 100 mW [39]. However, they are inefficient and require high power pumping sources. Quantum cascade lasers (QCLs) are originally developed in mid- and far-infrared spectral regions. The first THz QCL was demonstrated at 4.4 THz in 2002 [40]. THz QCLs are unipolar semiconductor lasers relying on intersubband transitions in GaAs/AlGaAs quantum wells which can be engineered to produce the desired wavelength of emitted radiation. Such lasers are very compact, but it is difficult to tune their frequency over a broad band. Moreover, mostly they work at low temperatures and need a cryogenic cooling system.

At present, a popular photonics-based approach for CW THz generation is to use photomixing [9], where two lasers with closely spaced emission frequencies are ‘mixed’ in an ultrafast semiconductor photodetector (PD) or PC antenna, generating a photocurrent with a frequency that is equal to their frequency difference in the THz region. This photocurrent is then used to drive an antenna and the result is the emission of THz waves. Photomixing has the advantage that the frequency of the THz waves can easily be tuned by adjusting the spacing of the lasers. Suitable photomixer devices include PC antennas [41], uni-travelling-carrier photodiodes (UTC-PDs) [42], travelling-wave uni-travelling-carrier photodiodes (TW-UTC-PDs) [43], or n-i-p-n-i-p superlattice photomixers [44]. However, the efficiency of optical-to-THz conversion is low, which limits the output power of most CW THz signals to the microwatt level [9,45]. The emission in a UTC-PD is typically at frequencies up to 1.5 THz. The maximum CW output power of 10.9 μW has been achieved at 1.04 THz with good linearity, pumping by laser diodes operating at 1.55 μm [46]. It is a common device used in current THz wireless communication systems. Of course, these technologies are still evolving rapidly, and the latest work compares the performance of two typical commercially available photomixers: UTC-PD and PIN-PD [47]. The UTC-PD emits ~100 μW at 250 GHz, and the PIN-PD produces 30 μW. Apart from the PD or PC antenna, another photonics-based way of generating CW THz radiation is to exploit a nonlinear medium in which incident electromagnetic waves undergo nonlinear frequency conversion [14]. Tunable lasers and photomixers are very important for CW THz generation. The big advantage of CW THz generation vs. broadband THz pulse generation is its simple optical source. Only two single-mode CW lasers are needed, with at least one of them tunable. Especially at the telecom wavelength of 1.55 µm, a large variety of compact, tunable, and low-cost semiconductor lasers are already available.

## 3. Detecting THz-Waves through Photonic Approaches

THz detection schemes fall into two general categories: coherent and incoherent techniques. The fundamental difference is that coherent detection measures both the amplitude and phase of the field, whereas incoherent detection only measures the intensity. Coherent detection techniques are closely associated with generation techniques in that they share underlying mechanisms and key components. In particular, optical techniques utilize the same light source for both generation and detection. Corresponding to the THz generation, it can also be divided into pulsed and CW detection.

### 3.1. Detection of Broadband THz Pulses

Sensing with a PC antenna or electro-optic (EO) sampling techniques can measure the actual electric field of broadband THz pulses in the time domain [2]. In the absence of a bias field, a THz field induces a current in the PC gap when an optical probe pulse injects photocarriers. The induced photocurrent is proportional to the THz field amplitude. The THz pulse shape is mapped out in the time domain by measuring the photocurrent while varying the time delay between the THz pulse and the optical probe. Alternatively, EO sampling techniques are based on the Pockels effect. A THz field induces birefringence in a nonlinear optical crystal which is proportional to the field amplitude. The entire waveform is determined by a weak optical probe measuring the field-induced birefringence as a function of the relative time delay between the THz and optical pulses.

**Figure 1:**
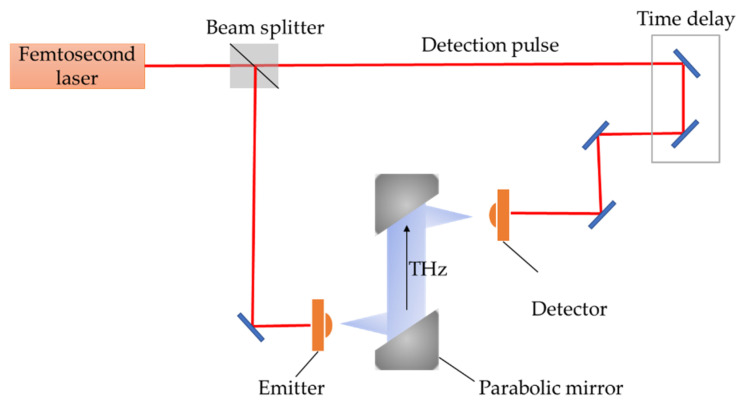
Schematic of a typical setup for generation and detection of THz pulses using femtosecond optical pulses.

The basic experimental setup for the generation and detection of THz pulses using a femtosecond laser is similar to the pump-probe technique. The schematic of the typical setup is shown in Figure 1. The principle of THz-TDS starts with a femtosecond laser producing an optical-pulse train. The optical beam is split into two paths. One illuminates the THz emitter, such as a PC antenna or nonlinear crystal, where the THz pulses are generated. These then propagate in free space and are focused onto a probe-pulse-gated detector, such as a PC antenna or an EO crystal. The other part of the pulse is also delivered onto the detector after passing through a time-delay stage. A 10-fs-laser allows the components of waves exceeding a frequency of 100 THz to be detected either by EO sampling [19] or a PC antenna [20]. In this ultraband coherent detection system, the scanning range and speed of the time-delay stage are important.

### 3.2. Detection of THz Continuous Waves

**Figure 2:**
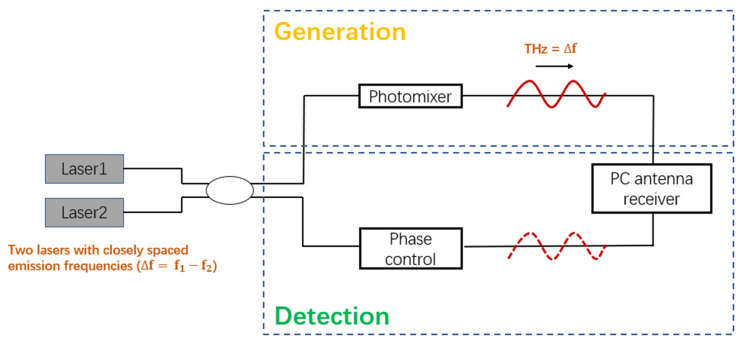
Schematic of a typical setup of photonics-based CW THz system.

In comparison with the THz-TDS system, photonics-based CW THz systems can use compact, low-cost, and well-developed fiber-optic telecom technologies. In general, a CW THz system has high-resolution frequency selectivity in a wide frequency range [48,49,50]. The coherent detection technique measures CW THz radiation by exploiting a PC antenna or EO sampling receiver. Figure 2 illustrates the commonly used coherent detection scheme of CW THz systems based on PC antenna. In this case, the photocurrent shows sinusoidal dependence on the relative phase between the optical beat and the THz radiation. In addition, in the high-speed THz communication systems, heterodyne detection is usually used to detect CW THz radiation based on a nonlinear device called a “mixer”. Schottky diodes are commonly used as mixers [4,51]. The key process in a mixer is frequency downconversion, which is carried out by mixing a THz signal with reference radiation at a local fixed frequency. The mixer produces an output signal at the difference frequency called the “intermediate frequency”. The amplitude of the output signal is proportional to the modulated signal amplitude.

Other methods are incoherent detections of CW THz radiation which means that they are not phase-sensitive and only provide signal amplitude detection. These detection methods usually no longer rely on optics, but mainly use thermal and electronic methods. Commonly used thermal detectors for observation of CW THz radiation are bolometers, Golay cells, and pyroelectric devices [52]. Because thermal conversion is generally wavelength-independent, the measured signals do not depend on the spectral content of the radiant power. Hence, thermal detectors can be very broadband detectors. More sophisticated thermal detectors are developed to achieve high sensitivity, such as microelectromechanical systems with a metamaterial absorber operating at the desired frequency (3.8 THz) [53]. All thermomechanical systems have slow-speed responses and are difficult to be integrated. Various electronic detectors based on rectification processes of electromagnetic signals such as Schottky diodes can be used in incoherent detection too [51,52,54]. All these rectification type detectors are fast, mostly only limited by the parasitic elements and readout electronics. Hence, they are widely used in THz communication systems.

## 4. Silicon Photonics for THz Techniques

**Figure 3:**
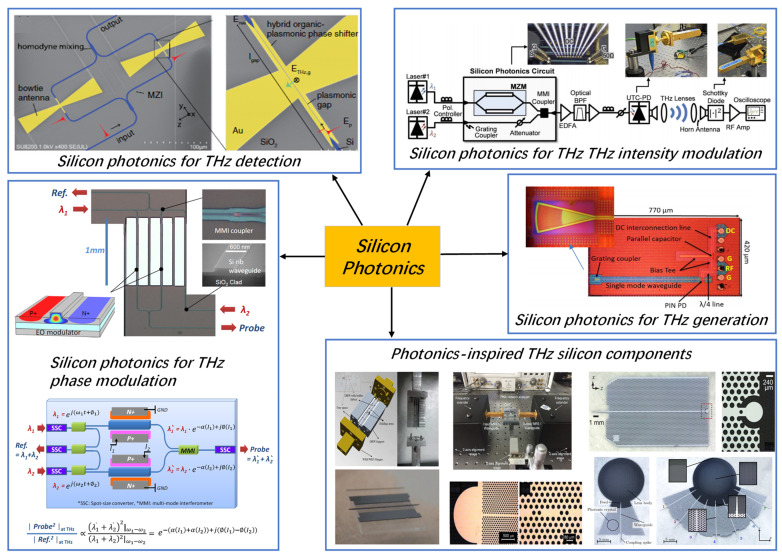
Applying silicon photonics in the THz techniques. © 2018 IEEE. Reprinted, with permission, from [55]. Reprinted with permission from [56]. © The Optical Society, 2020. Reprinted with permission from [57]. © The Optical Society, 2014. Reprinted with permission from [58]. © The Optical Society, 2020. Reprinted with permission from [59]. © The Optical Society, 2017. Reproduced from [60], with the permission of AIP Publishing, 2018. Reproduced from [61], with the permission of AIP Publishing, 2009. © 2014 IEEE. Reprinted, with permission, from [62]. © 2015 IEEE. Reprinted, with permission, from [63]. Reprinted with permission from [64]. © The Optical Society, 2018.

**Figure 4:**
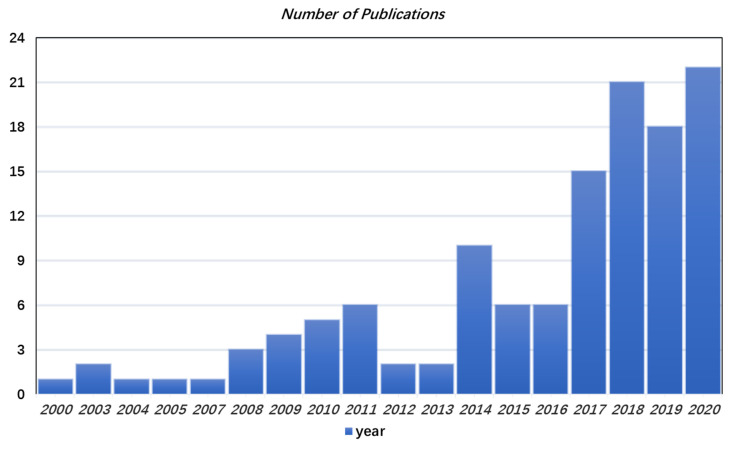
The number of publications of THz technology based on silicon photonics per year as obtained from Web of Science, starting from the year 2000.

Having provided how photonics technology is capable of realizing the THz functionalities, we now discuss the implementation of these functionalities on silicon photonics or hybrid silicon photonics platform. As explained in the introduction, the silicon photonics platform provides the highest level of fabrication maturity in terms of cost, yield, reproducibility, and standardization, which is crucial in the commercialization of photonics-based THz devices. Besides, the co-integration of electronic and photonic circuits offers distinct advantages over co-packaging approaches in the THz-wave regime, such as higher attainable speeds and lower power consumption [3]. The silicon-based devices that have been applied in the field of THz techniques mainly include signal generation, detection, phase modulation, and intensity modulation, as shown in Figure 3. Especially in signal generation, the advantage of the integrated scheme is that the on-chip beam steering can be realized or the total power of the radiation source can be increased through the device array. Figure 4 shows the number of publications of THz technology based on silicon photonics per year as obtained from Web of Science, starting from the year 2000. It shows the growing trend of this field. With the development of silicon germanium (Ge) technology and silicon hybrid integration with III-V materials, excellent performance will be expected for the application of silicon photonics technology in the THz field. The main photonic building blocks and device topologies that enable photonics-based THz systems are not much different from those needed in photonic integrated circuits.

### 4.1. Silicon Photonics for THz Generation

According to the principle introduced in Section 2, the key components of THz generation include lasers, ultra-high-speed optical detectors, or photomixers. While Si does not have a direct bandgap, thus lacking the ability to have light amplification through stimulated emission and achieve on-chip lasing, this issue can be resolved by the hybrid or heterogeneous integration of active III–V materials on silicon-on-insulator (SOI) using e.g., flip-chip packaging or wafer-bonding. Commonly used integrated lasers fall into two categories: one is the CW laser and the other is the mode-locked laser.

#### 4.1.1. CW Lasers

**Figure 5:**
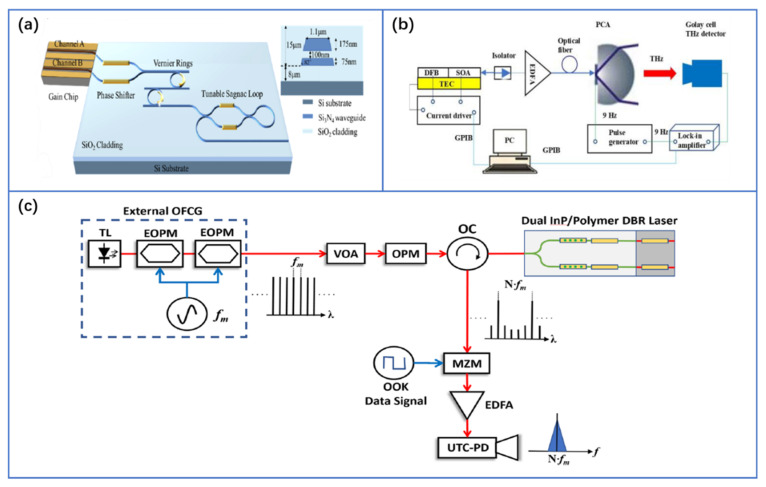
(**a**) Schematic view of a tunable InP-Si_3_N_4_ hybrid ECL with dual-parallel gain. Reprinted with permission from [76] © The Optical Society, 2021. (**b**) Setup for the THz generation and detection system based on the dual-mode DFB semiconductor laser. Reproduced with permission from J. H. Marsh, 2018. (**c**) Schematic of the experimental setup for optical injection locking of the hybrid dual InP/Polymer DBR photonic integrated circuit for THz carrier wave generation. Reproduced with permission from [87], Copyright, Springer Nature, 2018.

The common approach of optical heterodyne THz source is to integrate two CW lasers on the same substrate and combine their outputs, following the same scheme used with discrete lasers. As explained previously, the major drawback is that the traditional SOI platform does not contain monolithically integrated lasers. Thus, direct bandgap III-V materials provide a realistic solution for the integrated CW laser, but their integration on silicon is not straightforward. Heterogeneous approaches are desired for the monolithic platform, and tremendous progress has been made using quantum dots as gain materials [65,66,67,68,69,70]. However, the low-loss coupling of the light from III–V to silicon photonic circuits on the same chip is still a major obstacle. The more commonly used approaches are hybrid integration of silicon and III–V, where the III–V laser is flip-chip packaged or assembled with the silicon photonic chip [71,72,73,74,75,76,77]. The common requirements for the laser to generate narrow and stable THz radiations are high power, wide tuning range, and low frequency (linewidth) and phase noises between two CW modes. In the past decade, a linewidth reduction of four orders of magnitude is achieved on hybrid and heterogeneous platforms. Hence, the linewidth of heterogeneously integrated and hybrid integrated lasers can reach a better target than traditional monolithic III–V semiconductor lasers, approaching sub-kHz levels [78]. For example, a heterogeneous III–V/Si laser configuration containing long low-loss Bragg gratings improves the lasing stability and on-chip power up to 37 mW and a low linewidth of 1 kHz was demonstrated, although it had little wavelength tunability [79]. Recently it has been reported that silicon nitride external cavity lasers have achieved 6.6 kHz linewidth under on-chip output power of 23.5 mW with full C-band wavelength tunability [76], as shown in Figure 5a. To achieve higher optical power and keep the noise figure at the minimum, a hybrid III–V silicon nitride laser has achieved a record on-chip power of up to 20.7 dBm, a tuning range of 120 nm, and an ultralow linewidth of 320 Hz [80]. Although the existing integrated lasers which have been used for THz generation are mostly based on III–V distributed feedback (DFB) and distributed Bragg Reflector (DBR) lasers [81,82,83,84,85,86,87], as shown in Figure 5b,c, it can be predicted that hybrid and heterogeneously integrated silicon lasers with higher performance have the potential to achieve the target metrics and extend their applicability in THz band.

#### 4.1.2. Mode-Locked Lasers

**Figure 6:**
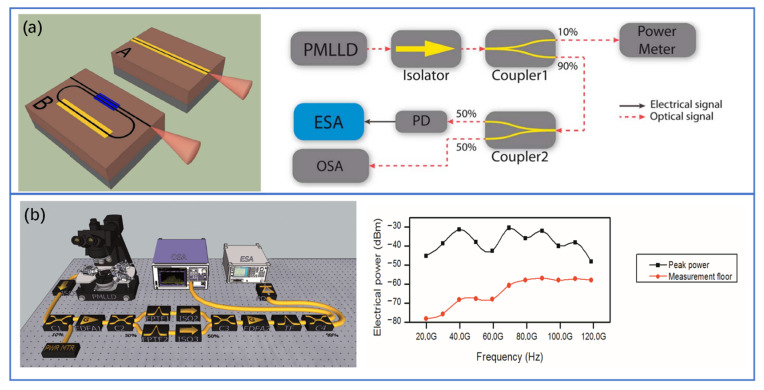
(**a**) Schematic view of laser cavities and experimental setup for electrical signal characterization. © 2012 IEEE. Reprinted, with permission. from [91]. (**b**) One CW sub -THz photonic synthesis setup and electrical peak power (black squares) and measurement floor (red circles) of the electrical signals. Reprinted (adapted) with permission from [93] © The Optical Society, 2012.

Mode-locked lasers (MLLs) have spectrally equidistant comb lines whose phases are locked to generate a pulsed output, where the repetition rate of the pulses is equal to the frequency difference between two adjacent comb lines. The femtosecond laser mentioned in the THz-TDS above is an MML. Fully integrated MLLs with III-V materials (InP, GaAs, etc.) as gain and slow saturation absorbers have been implemented for many years. The pulse width is in the order of picosecond. III-V gain sections have been recently combined with silicon photonics via heterogeneous integration [88,89,90]. The overview schematic of the MLL architecture is given in Ref. [89]. The peak power of the existing silicon-based MLLs is generally less than 1W [89,90]. Although the peak power can be improved by appropriately reducing the repetition frequency, the input peak power of the THz-TDS PC antenna is usually at the level of kW, and hence the output power of integrated MLLs still needs to be greatly improved. In addition, the pulse width of fully integrated MLLs is usually in the ps order (frequency comb width is narrow). To expand the bandwidth of THz pulses, the MLL pulse duration needs to be no more than 100 fs, so the properties of current integrated MLLs do not have high enough peak power or frequency comb bandwidth to be outright competitive with their bulk and fiber laser counterparts. Therefore, the current silicon-based integrated MLLs are difficult to be used in the THz-TDS system. Nevertheless, integrated MLLs can be used well for CW THz generation which relies on the combination in a photomixer device of two optical frequencies. To select two modes from the optical spectrum through filtering or other methods, the THz signals generated through optical heterodyne are phase-locked. This method is more suitable to generate low noise and narrow THz signals than two independent CW sources. The scheme of integrated MLL based on InGaAsP/AlGaInAs and InP has been used for THz signal generation [91,92,93,94,95]. Figure 6a,b show two examples of the CW THz photonic synthesis scheme. Values for the peak power of the electrical signals as well as the measured noise floor at each synthesized frequency are represented in Figure 6b. The photodiode used in this experiment has a 3-dB bandwidth of 50 GHz and it limits the performance in the THz band. This experiment can be readily scaled to synthesize higher frequencies by using UTC-PDs, etc. These schemes verify the feasibility of the integrated MLLs for THz signal generation. Hence, we can deduce that, in the near future, the THz signal generation based on mode-locked lasers can be realized on the SOI platform by using heterogeneous or hybrid silicon-based integration technology [94].

#### 4.1.3. High-Speed PDs and Photomixers

Optical heterodyning enabling the generation of microwave signals was reported as early as 1955 [95]. In practice, the frequency tuning range is typically limited by the PD or PC antenna bandwidth. Therefore, in addition to the above research on the silicon-based optical source, another key component used for THz signal generation is ultra-high-speed PDs or photomixers. The current high-speed silicon-based PDs or photomixers available for THz technology can be divided into roughly three groups: Ge PC antennas or PDs on silicon, new 2-D materials such as graphene, and nonlinear effect of metal surface plasmon polaritons.

**Figure 7:**
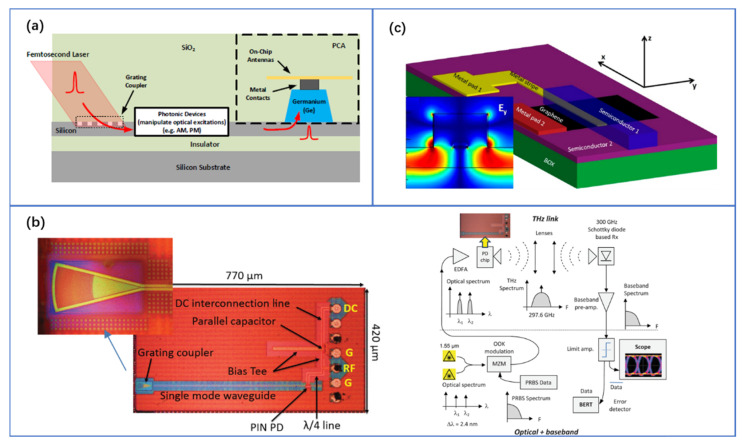
(**a**) Conceptual illustration of a waveguide-coupled THz photoconductive switch integrated on the SOI platform. Reproduced with permission from [97], Copyright, MDPI, 2019. (**b**) 220–330-GHz SiGe PD test structure and experimental setup for THz communication link. © 2012 IEEE. Reprinted, with permission, from [63]. (**c**) Schematic of the graphene photodetector with a 500 GHz bandwidth. Reprinted with permission from [109]. Copyright, American Chemical Society, 2020.

One common photomixer for achieving THz sources is the ultrafast PC antenna based on LT-GaAs, which is possible due to their ultrashort carrier lifetime [9,11]. However, the high cost of these materials prevents their use in high-volume applications. Moreover, it is not compatible with the low-cost 1.55 μm lasers used in fiber-optic communications. Thus, an SOI PC antenna was proposed to generate the THz signal by coupling the femtosecond laser pulses into the silicon waveguide and then switching to the ion-implanted Ge waveguide [96,97,98]. Because Ge has a long photo-carrier lifetime, n-type dopants are implanted (dose: 4 × 10^15^ cm^−2^) to reduce the photo-carrier lifetime to sub-picosecond. This demonstration is illustrated in Figure 7a, where a Ge-based optical waveguide, integrated on the SOI platform and coupled to a THz photoconductive antenna, was fabricated. It can emit THz pulses with a bandwidth of 1.5 THz [96]. This novel integrated waveguide-coupled photoconductive antenna makes it possible to integrate the THz radiator with an on-chip laser. It also allows electronic beam-steering of THz pulses by delaying the optical excitation signal through an electrically driven optical delay line. Furthermore, by integrating optical delay components and photoconductive antennas on a single chip, the entire THz-TDS system can be replaced by a single pocket-sized module.

Besides being used in PC antennas, Ge can also be applied in the high-speed PDs on SOI chips. In IMEC’s platform, SiGe PDs with a bandwidth up to 50 GHz are available [99], while other platforms and implementations provide even larger PD bandwidths [100,101,102]. For example, a Ge PD, that was grown at the end of a Si waveguide and operates around a wavelength of 1550 nm, was demonstrated to have a bandwidth of 120 GHz [103]. If we go beyond the 3-dB bandwidth and compromise on conversion efficiency, even higher THz frequencies can be generated. A SiGe PIN PD can be used to produce the output power of −28 dBm at 200 GHz [104]. In addition, a 180 GHz compact silicon photonic optically driven radiator was reported, using a multiport traveling-wave antenna driven by 8 SiGe PDs with a 25 GHz bandwidth. It can boost the broadside effective isotropic radiated power to −9.7 dBm [105]. Based on the above technologies, existing studies have realized 300 GHz on-off keying communication of 10 Gb/s or even 20 Gb/s, using SiGe PDs, as shown in Figure 7b [55,106]. In the future, the radiation efficiency and bandwidth of the chip can be further optimized. The lens coupling can also be used to avoid the difficulty in the high-speed probe and waveguide coupling, and it is also easy to pack.

An alternative route to realize high-speed PDs or photomixers is to introduce other new materials, such as two-dimensional materials. As reported in [107,108], graphene-based PDs integrated on Si photonic structures have unique properties and advantages such as high responsivity over a broad spectral range and fast operation. Indeed, a recent study shows that the plasmonic graphene PD on the Si waveguide, which relies on the photo-thermo-electric effect with the additional advantage of bias-free operation, has the potential to achieve the optoelectronic bandwidth up to 500 GHz, as shown in Figure 7c [109]. As such, the hybrid SOI/graphene platform opens up new opportunities to improve the performance of PDs that are designed to operate at THz frequencies.

Recently, T. Harter and colleagues reported the generation of THz waves using a plasmonic internal-photoemission detector (PIPED) [110]. It can be realized on the silicon photonics platform, which can support optical-to-terahertz (O/T) conversion. The structure consists of a silicon nanowire waveguide that is constructed by a thin layer of gold on the left and a titanium layer on the right. Light at the telecom wavelength of 1.55 μm is coupled to the silicon waveguide core, leading to excitation of surface plasmon polaritons at both the Au-Si and the Si-Ti interfaces. The hot electrons and holes are generated by free-carrier absorption cross the silicon barrier, leading to a photocurrent from the Au to the Ti side. They have demonstrated sensitivities of up to 12 mA W^−1^ and fast device response to be used for THz generation. Optical injection from two lasers with different frequencies enables photomixing, allowing THz generation in the frequency range of 0.1–1 THz. The radiated output power reaches –55 dBm at 300 GHz. Like a conventional III–V PC antenna, the photocurrent depends linearly on optical power around a specific bias point, enabling the photomixing process for CW THz generation.

### 4.2. Si Photonics for THz Detection

Photonics-based THz detection is based on coherent detection mechanisms, which can be performed with similar or even identical devices as used for THz generation. The most widely used receivers are PC antenna or EO sampling techniques. However, THz receivers are still quite complex from a technical point of view. The response function of the PC antenna depends on the carrier lifetime of the semiconductor substrate material. To achieve a short carrier lifetime, low-temperature growth of III–V semiconductor substrates (for example, GaAs or InGaAs/InAlAs multilayer structures) are required [111,112]. However, they are not amenable to large-scale photonic integration. Based on the electro-optic effect, EO sampling techniques should provide higher speed using crystals such as LiNbO_3_ [113] or ZnTe [50]. This method requires large interaction lengths and phase matching due to the small photon-to-photon up-conversion efficiency. As an alternative to inorganic crystals, organic material with a very high Pockels coefficient, such as DAST, provides a path to enhanced THz detection performance. A wideband 15 THz response using organic EO polymers has been demonstrated [114]. Even so, this THz detection system still relies on bulky free-space optics. Its detection sensors are still too large to be integrated and combined with generation devices on a common chip.

**Figure 8:**
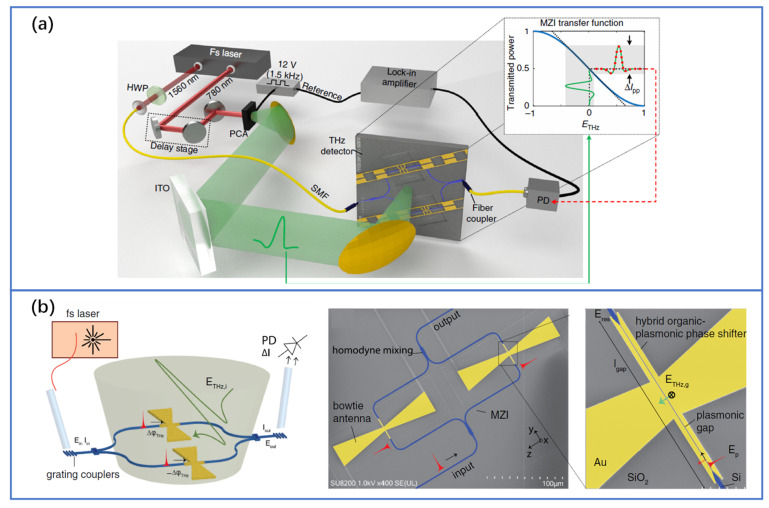
(**a**) THz time-domain EO sampling setup using the integrated plasmonic THz field detector consists of a Mach - Zehnder interferometer (MZI) with antenna-coupled plasmonic phase shifters. Reproduced with permission from [115], Copyright, Springer Nature, 2019. (**b**) THz time-domain EO sampling setup using an integrated detector with the bowtie antennas together with the layout of the detector. Reprinted (adapted) with permission from [56] © The Optical Society, 2020.

In order to develop the great application potential of THz systems, the monolithic integration of THz devices is very important. Although most of the current integrated THz research has focused on the generation, there are still some attempts in the detection of either pulsed or CW techniques. One can also employ the SOI platform for detection. Despite Ge has narrower bandwidth and lower sensitivity than InGaAs PC antennas, as we mentioned above, the silicon-based PC antenna has a lower cost of materials and can be integrated with lasers, which is still an important technology to be further developed and tried in the detection. Recently, integrated plasmonic waveguides have shown impressive mode confinement and can be combined with other materials, which offer a simple path towards integrated, efficient broadband THz detectors. For example, in terms of CW detection, the method based on internal photoemission at metal-semiconductor interfaces: the PIPED can also act as a THz-to-electrical (T/E) receiver, where a THz wave is mixed with the same optical reference used for O/T conversion [110]. It can receive radiation at frequencies up to 1 THz and exploit an intrinsic voltage-dependent sensitivity. To sample the THz pulses, ultra-compact integrated plasmonic THz field detectors are realized on a silicon platform [56,115]. The detectors consist of a Mach-Zehnder interferometer (MZI) with antenna-coupled plasmonic phase shifters, as shown in Figure 8a. The high efficiency is related to the strong confinement of the THz field and optical signal in the plasmonic waveguide with nonlinear organic material and a multi-resonant antenna design. In this way, an EO bandwidth of 2.5 THz with a dynamical range of 65 dB at an optical probe power of 63 nW was achieved [115]. The bowtie antennas were then used to improve the EO coupling rate and exhibit a > 70 dB dynamic range, as shown in Figure 8b [56]. This research further proves the possibility for fully monolithic THz systems operating at low power.

### 4.3. THz Phase Modulation

A homodyne detection scheme in the CW THz spectrometer system requires phase rotation of THz waves for the acquisition of a complete waveform. By varying the phase relationship between the incoming THz wave and the optical beat signal at the detector, both amplitude and phase of the detected THz signal can be recovered. Hence, a variation of phase in the THz or optical beam path is needed, as shown in Figure 2. The phase of the THz wave is determined by the phase difference of two lightwaves used in the photomixing [116]. When the phase of one of the lightwaves in the transmitter path is modulated, the output phase of the THz wave can be modulated. Using a delay stage is a simple way [117,118]. It can be used in the transmitter path or receiver path. The main problem of this simple implementation is that the velocity of the mechanical delay stage is slow. Moreover, it requires a free optical beam and thus prevents compact all-fiber implementation. Fiber stretchers can be used for rapid phase modulation. In a dual-stretcher configuration setup, two identical fiber stretchers are included in the transmitter and receiver arm [48,119]. It can reduce the sensitivity to thermal drifts and doubles the modulation amplitude compared to a single stretcher setup. Each fiber stretcher consists of several tens of meters of polarization-maintaining single-mode fiber wound around a piezoelectric actuator. The piezoelectric actuator is driven by an AC voltage to change the length of the fiber and thus the optical phase-modulation. Modulation of the optical path difference of 3 mm is obtained for a modulation frequency of ~1 kHz, which is much higher than a mechanical delay stage. A spectrum up to 1.8 THz with a 1 GHz step size is measured in only 10 min [48]. Therefore, the main advantage of this THz setup is the enhanced measurement speed and an all-fiber setup. Using the EO modulator is another possible compact all-fiber setup [49,120,121,122,123]. In these setups, the phase variation is electronically achieved by using an optical phase shifter instead of a path length change. Applying voltage in the EO modulator changes the refractive index and thus the optical phase. The advantages of using an optical phase modulator for phase-shifting are the elimination of the phase control speed limitation and the use of a more compact all-fiber setup. Besides, there is no free space part that generates radiation loss.

**Figure 9:**
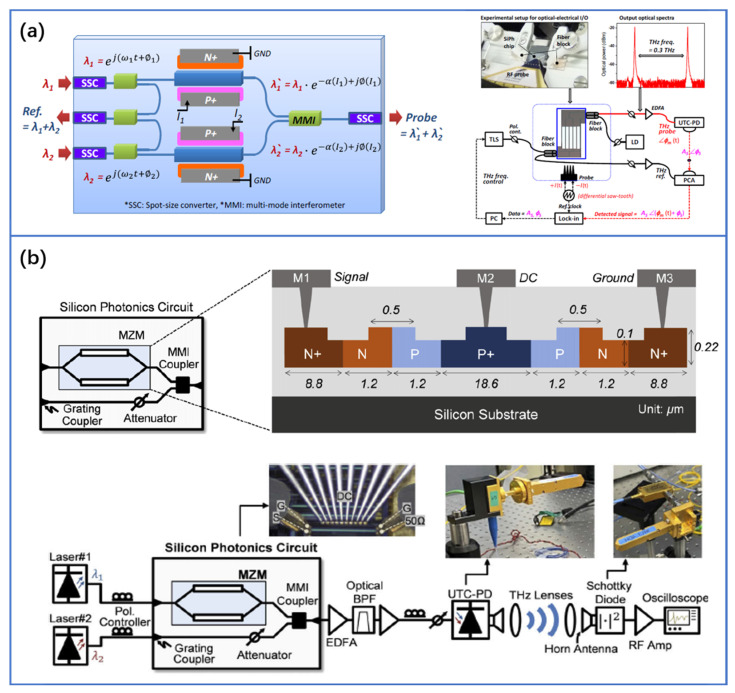
(**a**) Schematic diagram of photonic-THz phase control circuit integrated on the SOI photonic platform and experimental setup for CW THz homodyne spectroscopy system using silicon photonics circuit. Reprinted with permission from [56] © The Optical Society, 2014. (**b**) Schematic diagram of photonic-THz intensity modulation circuit integrated on the SOI photonic platform and experimental setup for the THz wireless communication system. Reprinted with permission from [58] © The Optical Society, 2020.

Although using EO modulators or fiber stretchers enables fast THz phase control without the physically moving parts. However, fiber-optic components are still too bulky, and the long fiber length can seriously degrade the phase stability of systems in vector detection. The THz phase control circuit, including EO modulators, couplers, and waveguides, can be monolithically integrated on a few square millimeters area of SOI [57,124,125]. As shown in Figure 9a, the phase control circuit consists of high-speed carrier-injection EO modulators with a balanced configuration to reduce the serious intensity distortion from each modulator. This phase control circuit based on silicon photonics can be used to enhance the phase stability of the system, resulting in a phase error of smaller than ± 10 degrees per 2 h. This demonstration shows the possibility of silicon photonics for THz phase modulation in a compact THz spectrometer and its huge potential for out-of-laboratory applications. Additionally, a fully integrated THz spectrometer will be the next step, using monolithic silicon photonics technology comprising a laser diode, high-speed PD, or PC antenna. This can improve the performance of the CW system for practical THz applications.

### 4.4. THz Intensity Modulation

The unallocated THz spectrum is gaining rising interest from future wireless communication networks thanks to its inherently advantageous properties such as broad bandwidth (at least tens of GHz) [4,51,126,127]. Photonics-based techniques offer the unique opportunity to ensure large BL products (where B is the bit rate and L is the wireless distance) in the THz wireless communication system [128,129,130,131,132]. For example, a photomixing generated 0.3 THz carrier transmits a record data rate beyond 100 Gb/s over 110 m through the simultaneous use of low-noise amplifiers (LNA) and power amplifiers (PA) [132,133]. In most THz photonic wireless links, the focus is on realizing stable integrated CW laser to generate high-purity THz carriers, while leaving the fast modulation to the modulator part, because the direct modulation technology is ultimately limited by its modulation bandwidth [134,135]. In the THz transmitter, one of the CW tones is modulated and the other one acts as an optical local oscillator, and they are combined for photomixing. An electrical signal with a frequency equal to the frequency difference of the two optical waves is generated. This technique is capable of generating an electrical signal from the microwave to the THz band, limited only by the bandwidth of the photomixing devices. As shown in Figure 9b, Sang-Rok Moon et al. have demonstrated a silicon-based photonic integrated circuit for optical modulation and coupling in the THz communication system [58]. They have performed experimental verification to show the feasibility of 40 Gb/s non-return-to-zero (NRZ) on-off-keying signal transmission over a 1.4 m THz wireless link centered at 300 GHz. In addition, since the THz photonic wireless system requires optical amplification before photomixing, this chip could be integrated with semiconductor optical amplifiers (SOA).

### 4.5. Photonics-Inspired THz Silicon Components

**Figure 10:**
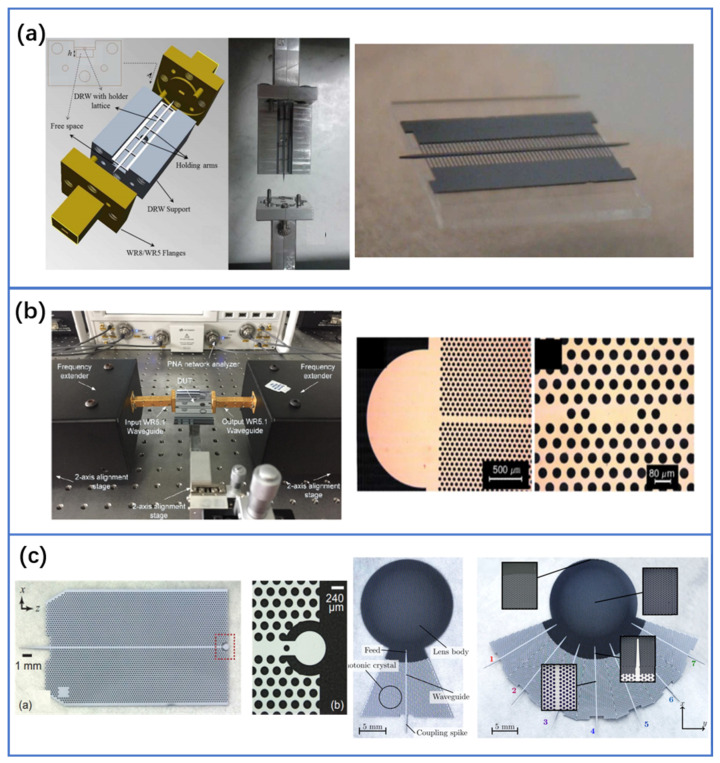
(**a**) THz silicon waveguides. © 2014 IEEE. Reprinted, with permission, from [62]. © 2015 IEEE. Reprinted, with permission, from [63]. (**b**) THz high-Q silicon cavities. Reproduced from [61], with the permission of AIP Publishing, 2009. Reprinted with permission from [64] © The Optical Society, 2018. (**c**) THz antennas implemented on silicon. Reprinted with permission from [59] © The Optical Society, 2017. Reproduced from [60], with the permission of AIP Publishing, 2018.

In the context of THz passive devices, it should be noted that there is growing research on the recently developed all-dielectric THz components implemented on silicon [59,60,61,136,137]. The high-resistivity silicon is an effective material in the THz band, due to its lower dispersion and loss characteristics [138]. Especially at higher frequencies, metal-based transmission lines and passive components inevitably have an ohmic loss. Therefore, a silicon platform is suitable for THz circuits to achieve simplicity and efficiency [139]. In addition to the THz silicon waveguides [62,63,140,141,142,143], many other passive functionalities for THz waves have been realized, such as high-Q silicon cavities [61,64,144,145,146], antennas [59,60], time-domain signal processing [147], and so on. To further reduce the waveguide loss, various waveguide structures are designed (see Figure 10a). A 19.7 mm silicon-on-glass waveguide is realized, and its propagation loss is very low. In particular, between 481.5 and 500 GHz, the measured loss is varying between 0.018 dB/mm and 0.104 dB/mm [63]. Compared with conventional planar metallic transmission lines, the loss of this waveguide is significantly reduced in the same frequency range. Metallic coplanar transmission lines exhibit attenuation in the order of several dB/cm [148,149]. Even using the low-loss dielectric as substrate, the attenuation is still high (about 3 dB/mm at 600 GHz) [150]. In order to validate the waveguide performance in THz communications, an effective medium cladded dielectric waveguide platform was experimentally characterized for its maximum transmission bit rate of 28 Gibt/s at 335 GHz [143]. An uncompressed 4K-resolution video transmission was also demonstrated. The high-Q cavities are fundamental to the implementation of compact sensors and filters [151]. As shown in Figure 10b, all-silicon THz cavities with Q as high as 2839 and 1020 have been realized [61,64]. Based on this, several tunable devices have been realized so far, such as thermal tuning of silicon THz whispering-gallery-mode resonators [152,153]. Antennas are vital for coupling guided waves in the THz silicon waveguides with free-space waves. There are several key factors to be considered when designing an antenna, including bandwidth, efficiency, radiation gain, and compactness. From the fabrication perspective, it is appealing to integrate the antenna with other THz silicon components on the same wafer. As an example, a dielectric resonator antenna (see the left inset of Figure 10c) can be fully integrated with a photonic crystal waveguide for end-fire radiation [59]. The experimental results confirm the 3-dB angular beam widths of 29.0 degrees and 45.7 degrees in orthogonal dimensions and the maximum gain of over 10.6 dBi. The photonic crystal waveguides can also be integrated directly with the Luneburg lens and fabricated together on the same silicon wafer (see the right inset of Figure 10c) [60]. The antenna gain was above 18 dBi over the operation bandwidth from 320 to 390 GHz. Cross talk of different channels was experimentally determined to be lower than −28 dB. These are important component developments for various THz applications, which can be readily scalable to higher frequencies. Besides, these planar silicon devices can be easily and monolithically fabricated using the CMOS process. It also means that in the future, silicon photonics platforms have great potential to implement an all-THz integrated circuit to some degree (in addition to the photonic components already offered), thus placing all the electronics, photonics, and THz functionality on one single chip for generating, detecting, and processing THz waves.

## 5. Summary and Outlook

Many key THz functionalities have been demonstrated on the SOI platform, with capabilities such as THz generation, detection, and modulation for applications in sensing, imaging, and communication. This integration technology can benefit from mature fabrication processes and reduce the size and cost of THz systems. Due to research and developments during the last two decades in the THz domain, photonics-based THz devices have already been employed in scientific laboratories and industrial research. The techniques of CW and pulsed THz systems have unique advantages and disadvantages that must be considered if they are to be used in different applications. By far, nonlinear photoconductors with a very short carrier lifetime, such as LT-GaAs, are still the most widely used for THz photomixing. However, their properties are difficult to control, and the fabrication processes are expensive. The SOI platform is a cheap, simple, and prospective option. One of the challenges faced by silicon photonics-based THz chips is related to energy consumption. Both the high-quality hybrid silicon lasers and efficient O/T and T/O conversions are required. Future work will be needed on the development of high THz output power and large bandwidth, in particular for applications such as long-distance transmission. In the long term, graphene-based or plasmonic-based technologies on the SOI platform will be a promising area of development, in particular for enhanced or broadband THz generation and detection. Finally, THz technologies can benefit further if low-loss planar waveguide technology is developed for on-chip THz interconnection and signal processing. The purpose of this paper is aimed at providing some inspirations after summarizing the current work, in the hope of promoting the further development of this field and attracting more researchers to pay attention to this direction.

## Data Availability

The study did not report any data.
